# Supplementary material for: Identification of photoperiod-induced specific miRNAs in the adrenal glands of Sunite sheep (Ovis aries)
Source: Front Vet Sci. 2022 Jul 22;9:888207. doi: 10.3389/fvets.2022.888207 (PMC9354845; doi:10.3389/fvets.2022.888207)
Supplement: Supplementary file 1 [file Data_Sheet_1.ZIP › Supplementary file/miRDeep2 file.zip!/miRDeep2 file/Know_Structure/SP42A3/oar-mir-30a.pdf]

miRBase precursor : oar-mir-30a
Total read count : 138286
oar-miR-30a-5p read count : 131730
oar-miR-30a-3p read count : 6555
remaining reads : 1

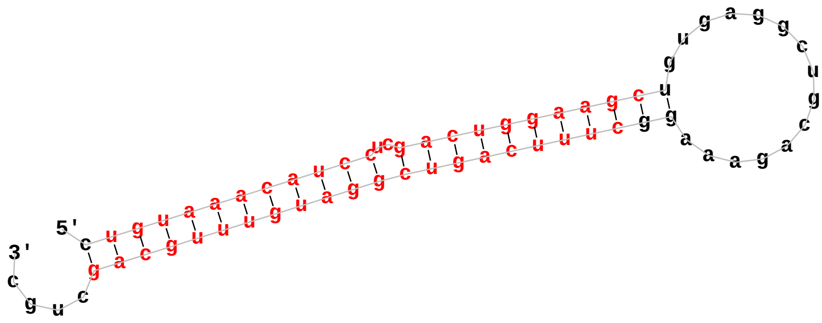

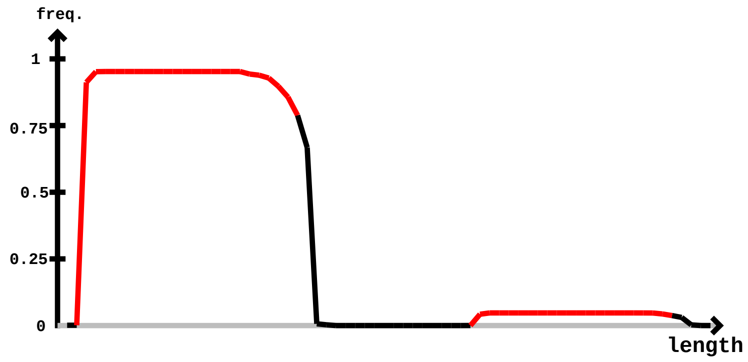


```
                                              oar-miR-30a-3p
  oar-miR-30a-5p
5'- cuguaaacauccucgacuggaagcugugaggcugcagaaaggcuuucagucggauguuugcagcugc   -3'    exp
    ((((((((((((..(((((((((((...............)))))))))))))))))))))))....    reads      mm         sample
    cuguaaacauccuUgacu.................................................    5          1          seq
    cuguaaacauccuUgacug................................................    1          1          seq
    cuguaaacauccucgacugg...............................................    3          0          seq
    cuguaaacauccuUgacugga..............................................    2          1          seq
    cuguaaacauccucgacugga..............................................    16         0          seq
    cuguaaacauccuUgacuggaa.............................................    2          1          seq
    cuguaaacauccucgacuggaa.............................................    14         0          seq
    cuguaaacauccuUgacuggaag............................................    3          1          seq
    cuguaaacauccucgacuggaag............................................    10         0          seq
    cuguaaacauccucgacuggaagc...........................................    39         0          seq
    cuguaaacauccuUgacuggaagc...........................................    1          1          seq
    cuguaaacauccuUgacuggaagcu..........................................    28         1          seq
    cuguaaacauccucgacuggaagcu..........................................    46         0          seq
    .uguaaacauccucgacu.................................................    176        0          seq
    .uguaaacauccuUgacu.................................................    1128       1          seq
    .uguaaacauccuUgacug................................................    432        1          seq
    .uguaaacauccucgacug................................................    160        0          seq
    .uguaaacauccucgacugg...............................................    773        0          seq
    .uguaaacauccuUgacugg...............................................    709        1          seq
    .uguaaacauccucgacugga..............................................    3543       0          seq
    .uguaaacauccuUgacugga..............................................    717        1          seq
    .uguaaacauccucgacuggaa.............................................    4762       0          seq
    .uguaaacauccuUgacuggaa.............................................    815        1          seq
    .uguaaacauccuUgacuggaag............................................    2833       1          seq
    .uguaaacauccucgacuggaag............................................    6562       0          seq
    .uguaaacauccuUgacuggaagc...........................................    2514       1          seq
    .uguaaacauccucgacuggaagc...........................................    14067      0          seq
    .uguaaacauccucgacuggaagcu..........................................    39080      0          seq
    .uguaaacauccuUgacuggaagcu..........................................    47465      1          seq
    .uguaaacauccuUgacuggaagcug.........................................    91         1          seq
    .uguaaacauccucgacuggaagcug.........................................    51         0          seq
    .uguaaacauccucgacuggaagcugu........................................    15         0          seq
    .uguaaacauccuUgacuggaagcugu........................................    59         1          seq
    ..guaaacauccuUgacugg...............................................    3          1          seq
```

```
                                        oar-miR-30a-3p
  oar-miR-30a-5p
cuguaaacauccucgacuggaagcugugaggcugcagaaaggcuuucagucggauguuugcagcugc

..guaaacauccucgacugga..............................................    1      0     seq
..guaaacauccuUgacugga..............................................    5      1     seq
..guaaacauccuUgacuggaa.............................................    4      1     seq
..guaaacauccucgacuggaa.............................................    4      0     seq
..guaaacauccucgacuggaag............................................    8      0     seq
..guaaacauccuUgacuggaag............................................    25     1     seq
..guaaacauccuUgacuggaagc...........................................    38     1     seq
..guaaacauccucgacuggaagc...........................................    24     0     seq
..guaaacauccuUgacuggaagcu..........................................    4448   1     seq
..guaaacauccucgacuggaagcu..........................................    307    0     seq
..guaaacauccuUgacuggaagcug.........................................    357    1     seq
..guaaacauccucgacuggaagcug.........................................    2      0     seq
..guaaacauccuUgacuggaagcugu........................................    301    1     seq
..guaaacauccucgacuggaagcugu........................................    1      0     seq
...uaaacauccucgacuggaagc...........................................    2      0     seq
...uaaacauccuUgacuggaagcu..........................................    23     1     seq
...uaaacauccucgacuggaagcu..........................................    19     0     seq
...uaaacauccuUgacuggaagcug.........................................    13     1     seq
...uaaacauccucgacuggaagcug.........................................    1      0     seq
...uaaacauccucgacuggaagcugu........................................    2      0     seq
...uaaacauccuUgacuggaagcugu........................................    20     1     seq
.........................gugaggcugcagaaagg.........................    1      0     seq
........................................AgcuuucagucggauguuuAc......    1      1     seq
.........................................gcuuucagucggauguuuAc......    1      1     seq
.........................................gcuuucagucggauguuuAca.....    10     1     seq
.........................................gcuuucagucggauguuuAcag....    4      1     seq
.........................................gcuuucagucggauguuuAcagc...    10     1     seq
..........................................cuuucagucggauguuu........    18     0     seq
..........................................cuuucagucggauguuug.......    5      0     seq
..........................................cuuucagucggauguuuA.......    13     1     seq
..........................................cuuucagucggauguuuAc......    386    1     seq
..........................................cuuucagucggauguuugc......    95     0     seq
..........................................cuuucagucggauguuuAca.....    730    1     seq
..........................................cuuucagucggauguuugca.....    75     0     seq
..........................................cuuucagucggauguuuAcag....    683    1     seq
..........................................cuuucagucggauguuugcag....    236    0     seq
..........................................cuuucagucggauguuugcagc...    1107   0     seq
..........................................cuuucagucggauguuuAcagc...    2407   1     seq
..........................................cuuucagucggauguuugcagcu..    99     0     seq
..........................................cuuucagucggauguuugcagcug.    1      0     seq
...........................................uuucagucggauguuuA.......    1      1     seq
...........................................uuucagucggauguuuAc......    22     1     seq
...........................................uuucagucggauguuugc......    3      0     seq
...........................................uuucagucggauguuuAca.....    8      1     seq
...........................................uuucagucggauguuugca.....    4      0     seq
...........................................uuucagucggauguuugcag....    12     0     seq
...........................................uuucagucggauguuuAcag....    24     1     seq
...........................................uuucagucggauguuugcagc...    157    0     seq
...........................................uuucagucggauguuuAcagc...    169    1     seq
...........................................uuucagucggauguuugcagcu..    259    0     seq
...........................................uuucagucggauguuugcagcug.    10     0     seq
............................................uucagucggauguuugcagc...    3      0     seq
............................................uucagucggauguuuAcagc...    1      1     seq
............................................uucagucggauguuugcagcu..    1      0     seq
```
